# 3D-printed PEEK versus conventional free fibula flap for jaw reconstruction: a comparative clinical study

**DOI:** 10.3389/fonc.2025.1656914

**Published:** 2025-10-29

**Authors:** Dongdong Feng, Yuxiang Zhang, Haiwei Guo, Chuanming Zheng, Yining Zhang, Wanchen Zhang, Fangyuan Lai, Jiajie Xu

**Affiliations:** ^1^ Otolaryngology and Head and Neck Center, Cancer Center, Department of Head and Neck Surgery, Zhejiang Provincial People’s Hospital, Affiliated People’s Hospital, Hangzhou Medical College, Hangzhou, China; ^2^ Key Laboratory of Endocrine Gland Diseases of Zhejiang Province, Hangzhou, China; ^3^ Clinical Research Center for Cancer of Zhejiang Province, Hangzhou, China; ^4^ Second Clinical Medical School, Hangzhou Normal University, Hangzhou, China; ^5^ The Second Affiliated Hospital of Zhejiang Chinese Medicine University, Hangzhou, China; ^6^ The First Affiliated Hospital of Ningbo University, Ningbo, China; ^7^ Center for Plastic and Reconstructive Surgery, Department of Plastic and Reconstructive Surgery, Zhejiang Provincial People’s Hospital (Affiliated People’s Hospital), Hangzhou Medical College, Hangzhou, China

**Keywords:** jaw, reconstruction, fibula free flap, PEEK, 3D printing technology

## Abstract

**Purpose:**

Aimed to evaluate the application value of polyether ether ketone (PEEK) based on three-dimensional (3D) printing technology for jaw reconstruction.

**Methods:**

This study retrospectively analyzed 16 patients who underwent jaw reconstruction in the Department of Head and Neck Surgery, Zhejiang Provincial People’s Hospital, from August 2019 to December 2023. A subsequent comparison was made between the effectiveness of free fibula flap (FFF) and PEEK schemes.

**Result:**

A total of 16 patients were included in this study. In the FFF group, six male and two female patients (mean age, 40.8 ± 21.2 years) underwent jaw defect reconstruction successfully. However, one patient in this group required the removal of the titanium plate due to uncontrollable infection. In the PEEK group, which consisted of five men and three women (mean age, 56.3 ± 12.6 years), six patients achieved satisfactory outcomes, including accurate jaw restoration and good occlusal function during follow-up, and were successfully discharged. However, two patients in this group experienced secondary infection, which necessitated the removal of the PEEK implants and subsequent salvage reconstruction using FFF. The operative time was significantly shorter in the PEEK group compared to the FFF group (*p* < 0.05). Infection was identified as the primary cause of reconstruction failure in the PEEK group, for which FFF served as an effective salvage procedure. Although the PEEK group showed lower means in intraoperative blood loss, postoperative drainage, and reduction in Body Mass Index (BMI) compared to the FFF group, these differences did not reach statistical significance.

**Conclusion:**

3D printing technology enables the fabrication of patient-specific PEEK implants with customized geometries. Owing to its stable biocompatibility, PEEK is suitable for reconstructing complex and irregular jaw defects. As a viable alternative for primary jaw reconstruction, it demonstrates promising clinical application prospects.

## 1 Introduction

The maxilla and mandible play a vital role in supporting facial morphology, mastication, speech, and other essential functions. However, their structure and function can be compromised by various factors, such as tumors, trauma, infection, and congenital deformities, which may severely affect patients’ quality of life and psychological well-being (1).

Materials used for jaw reconstruction are broadly categorized into bone grafts and non-bone materials. Bone grafts include autogenous bone, allogeneic bone, and xenogeneic bone, while non-bone materials comprise various metals and synthetic materials. The free fibula flap (FFF) is widely regarded as a reliable and effective option for jaw reconstruction and remains the most commonly used in clinical practice. Nevertheless, it has notable limitations, such as donor-site morbidity and limited flexibility in shaping the flap to match complex mandibular contours. Non-bone materials, in contrast, face constraints in clinical applicability due to challenges related to biocompatibility, fatigue resistance, and corrosion resistance (2).

The selection of an ideal material is critical for successful jaw reconstruction. Polyether ether ketone (PEEK), a polycyclic aromatic semi-crystalline thermoplastic polymer, exhibits excellent biocompatibility, fatigue resistance, mechanical properties, and radiolucency. Compared to other non-bony materials, PEEK possesses an elastic modulus (3–4 GPa) that more closely approximates that of cortical bone (approximately 18 GPa), whereas titanium (110 GPa) exhibits a significantly higher value. This favorable mechanical profile reduces stress shielding and promotes more physiological load transfer at the bone–implant interface (3). PEEK is already well-established in clinical applications, and the integration of 3D printing technology further enables the fabrication of patient-specific implants with customized geometries (4). Consequently, 3D-printed PEEK implants represent a promising approach for precise and individualized jaw reconstruction (5).

This study retrospectively analyzed cases of jaw reconstruction using FFF or 3D-printed PEEK implants with the aim of evaluating and comparing the clinical applicability and potential of PEEK in jaw reconstruction.

## 2 Materials and methods

### 2.1 Clinical data

We enrolled patients who underwent jaw defect reconstruction with either the FFF or PEEK protocol at the Department of Head and Neck Surgery, Zhejiang Provincial People’s Hospital, between August 2019 and December 2023. The detailed information of all enrolled patients is presented in **Table 1**. Reconstruction was indicated for both benign and malignant jaw lesions. In case of malignancy, concurrent cervical lymph node dissection was performed. For patients in the PEEK group with substantial soft tissue defects, reconstruction was supplemented using either a free flap or a local adjacent flap. Clinical data and prognostic information were systematically collected and analyzed for all participants. This study was approved by the Ethics Committee of Zhejiang Provincial People’s Hospital and was conducted in accordance with the principles of the Declaration of Helsinki.

**Table 1:** Baseline characteristics of patients.

Group	Code	Age	Gender	Disease	TNM	Diameter	Lymph node dissection	Radiotherapy	Follow-up time (months)
						(cm)			
FFF	F1	28	F	Pleomorphic adenoma of the right maxilla	——	4	——	——	49
	F2	57	M	Well-differentiated squamous cell carcinoma of the lower right gingiva	T4N0M0	3.5	Y	Y	55
	F3	18	M	Ossifying fibroma of the right mandible	——	3.8	——		39
	F4	25	M	Ameloblastoma of the right mandible	——	3.5	——		35
	F5	25	F	Ameloblastoma of the right mandible	——	2.5	——		50
	F6	34	M	Odontogenic ghost cell carcinoma of the right maxillary sinus	T2N0M0	3.5	Y		37
	F7	71	M	Moderately differentiated squamous cell carcinoma of the lower right gingiva	T2N0M0	2.5	Y	Y	36
	F8	68	M	Well-differentiated squamous cell carcinoma of the lower right gingiva	T3N0M0	5	Y	Y	36
PEEK	P1	48	M	Postoperative status of moderately differentiated squamous cell carcinoma of the lower left gingiva	T1N0M0	5	——	——	41
	P2	68	M	Cystic change of the left mandible	——	2.4	——	——	16
	P3	75	M	Moderately differentiated squamous cell carcinoma of the upper left gingiva	T2N0M0	2.5	Y	Y	38
	P4	61	M	Moderately differentiated squamous cell carcinoma of the floor of mouth	T2N1M0	3.8	Y	Y	7
	P5	64	M	Moderately to well-differentiated squamous cell carcinoma of the lower right gingiva	T2N1M0	3.9	Y	NA	53
	P6	38	F	Benign tumor of the left maxilla	——	3	——	——	12
	P7	46	F	Odontogenic benign tumor of the right mandible	——	1.5	——	——	41
	P8	50	F	Well-differentiated squamous cell carcinoma of the lower left gingiva	T4N0M0	4.1	Y	Y	52

NA, not available; Y, yes; FFF, free fibula flap; PEEK, polyether ether ketone.

### 2.2 Preoperative planning and operating procedure

**Figure 1:**
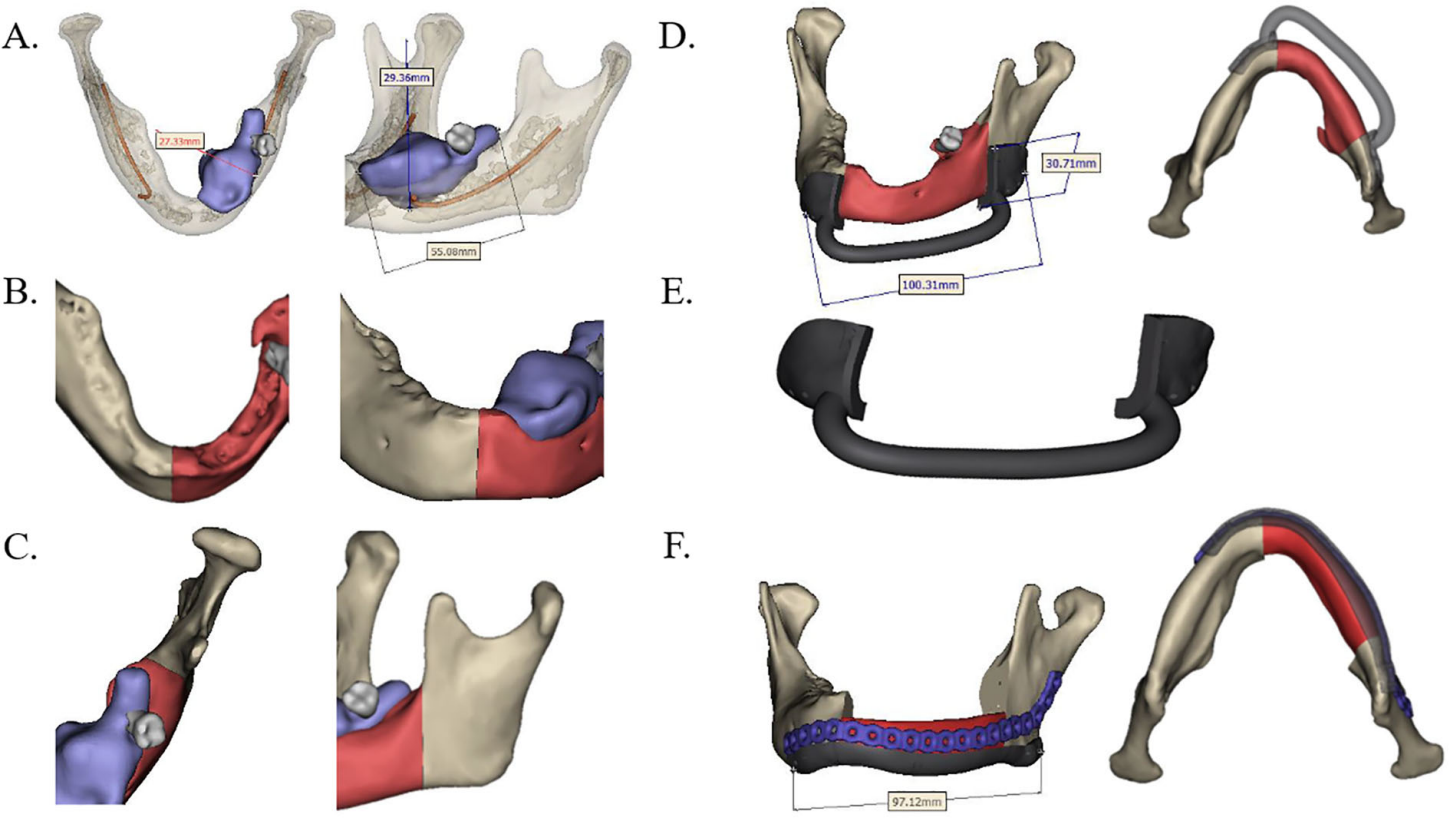
Design of preoperative jaw resection and PEEK reconstruction scheme. **(A)** Predetermined mandibular resection range based on contrast-enhanced CT scan data (highlighted red part). **(B, C)** Anterior and posterior margins of the planned resection range. **(D, E)** Osteotomy guide and patient-specific PEEK implant designed in accordance with the resection boundaries. **(F)** The PEEK implant (red) was positioned and fixed with a procurved titanium plate after resection. PEEK, polyether ether ketone.

**Figure 2:**
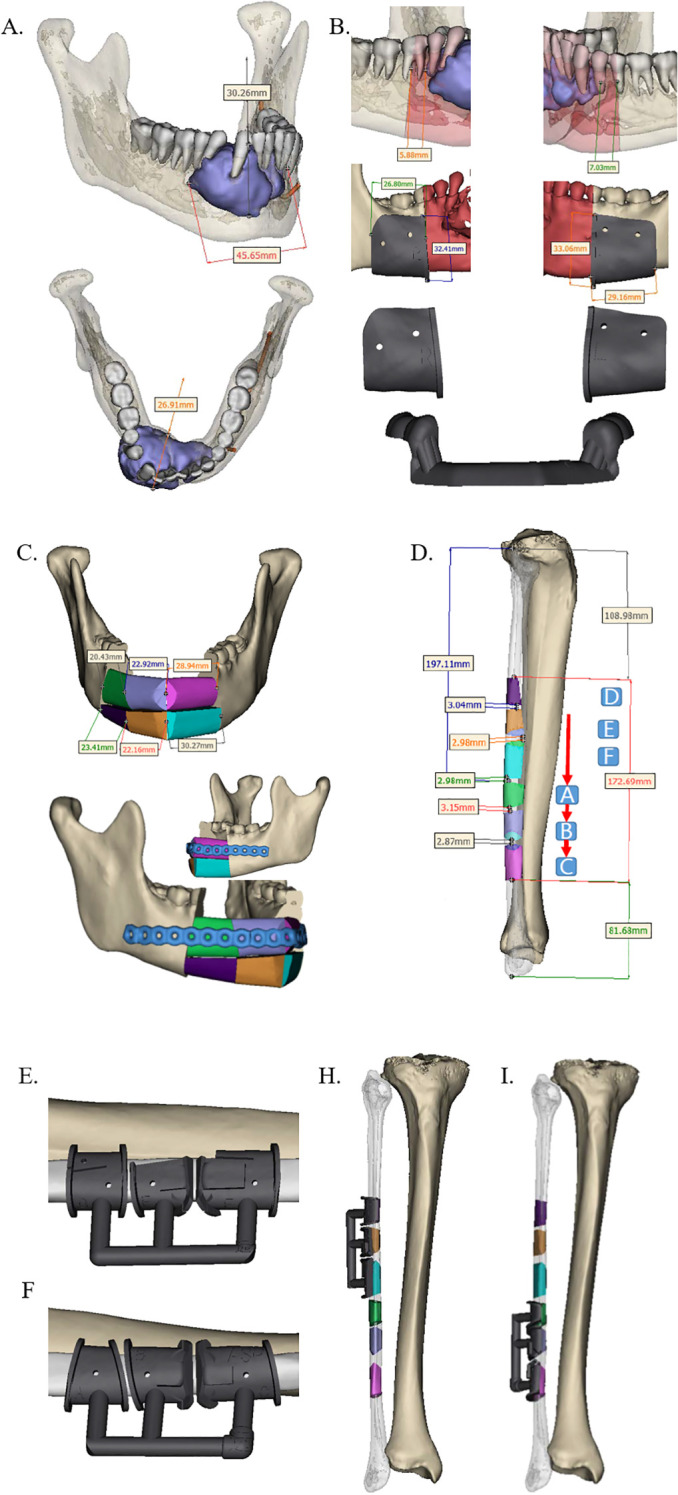
Design of preoperative jaw resection and FFF reconstruction scheme. **(A)** Predetermined mandibular resection margins based on contrast-enhanced CT data (lateral and superior views). **(B)** Anterior and posterior margins of the resection area, along with the corresponding osteotomy guide. **(C)** Design of the FFF reconstruction and the procurved titanium plate for fixation. **(D)** Precisely segmented fibula graft for mandibular reconstruction (color-matched to the scheme in panel **C**). **(E–I)** Intraoperative positioning of the osteotomy guide on the fibula. FFF, free fibula flap.

The extent of the lesion or defect was assessed using contrast-enhanced CT and MR, followed by three-dimensional (3D) reconstruction of the jaw. Based on the planned surgical resection margins, the geometry of both the FFF and the patient-specific PEEK implant—including procurved titanium plates (**Figure 1**)—was meticulously designed. In the FFF group, a fibular osteotomy was also preoperatively planned to achieve accurate reconstruction (**Figure 2**). Additionally, surgical osteotomy guides were designed to facilitate precise bone shaping during the procedure (**Figures 1E**, **2B**). All 3D reconstructions, PEEK implants, and osteotomy guides were designed and manufactured by a certified medical device company.

**Figure 3:**
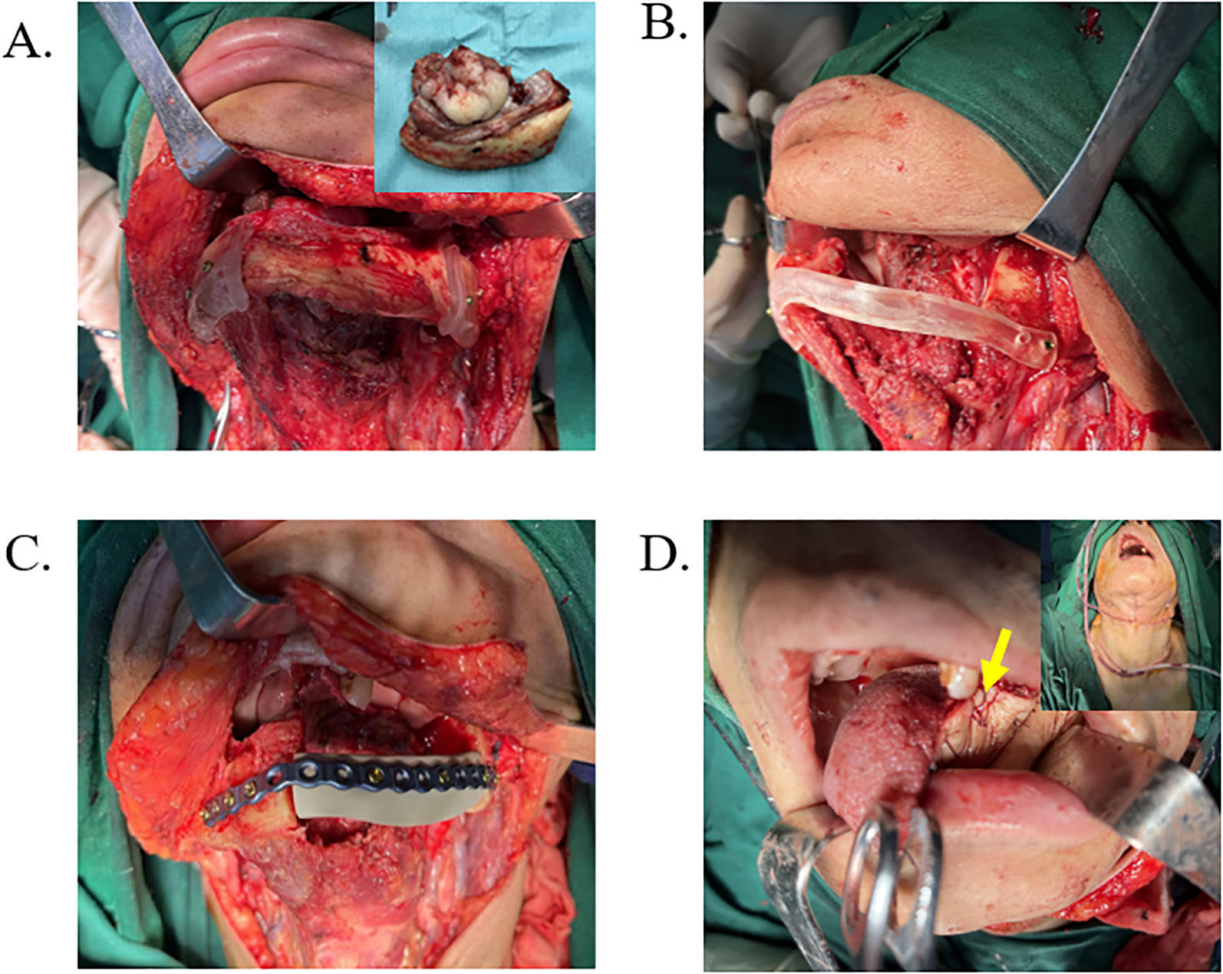
Surgical procedure based on PEEK reconstruction scheme. **(A)** Intraoperative exposure of the surgical site with positioning of the osteotomy guide, followed by removal of the lesion tissue. **(B)** The osteotomy guide supporting the mandibular defect area. **(C)** The PEEK implant was fixed in place using a procurved titanium plate. **(D)** Reconstruction of the soft tissue defect with a flap (yellow arrow), followed by drain placement and wound closure.

**Figure 4:**
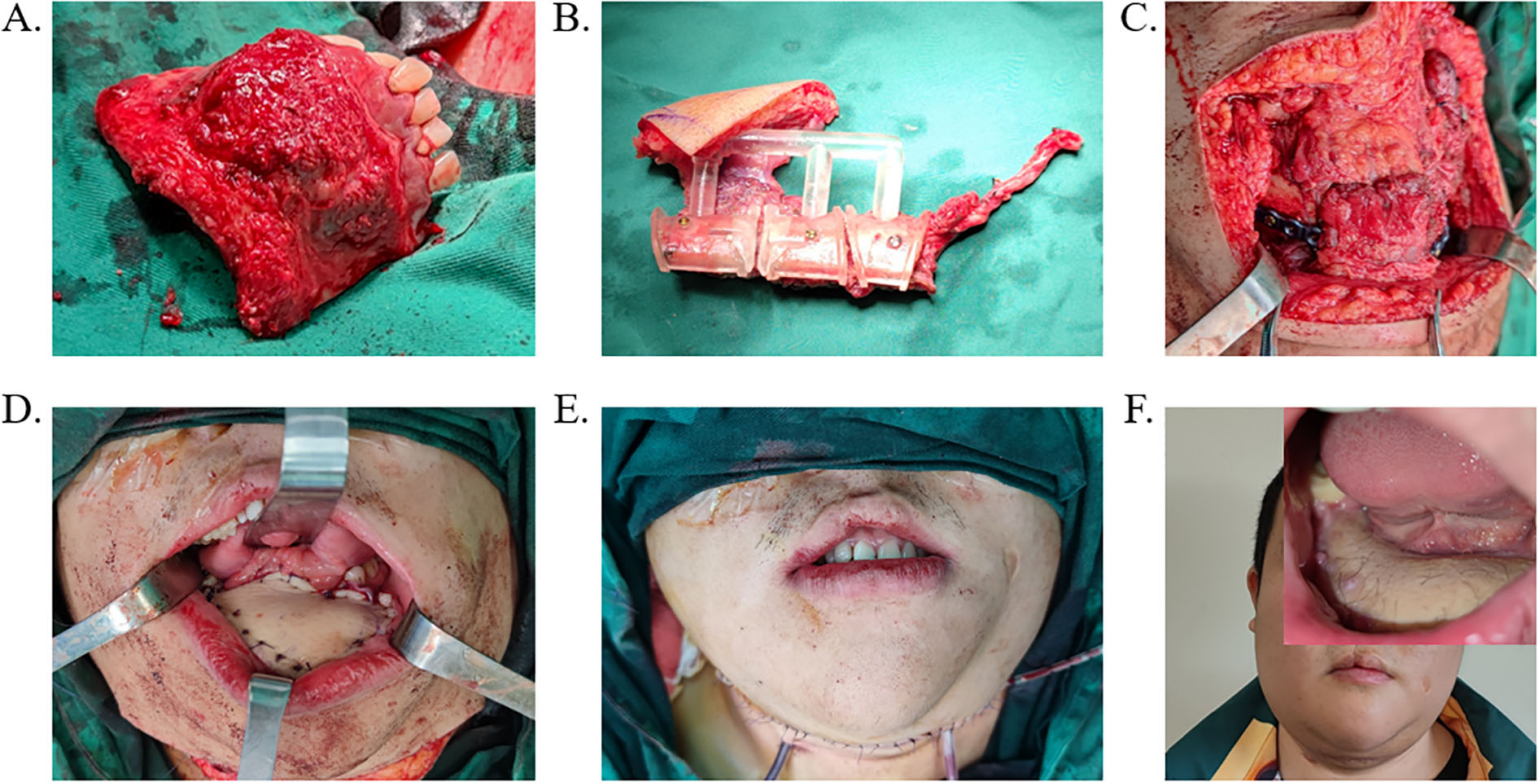
Surgical procedure based on FFF reconstruction scheme. **(A)** Resection of the lesioned area guided by the osteotomy template. **(B)** Osteotomy of the fibula performed using the surgical guide. **(C)** Implantation and fixation of FFF into the mandibular defect with a titanium plate. **(D, E)** Wound closure and drainage tube placement. **(F)** Postoperative follow-up outcome following FFF reconstruction. FFF, free fibula flap.

Following preoperative preparation, all surgical procedures were performed in the operating room of Zhejiang Provincial People’s Hospital. The PEEK implants and osteotomy guides were routinely sterilized 24 hours prior to surgery. In accordance with standard jaw reconstruction protocols, the surgical site was exposed, and the osteotomy guide was positioned to facilitate precise bone resection (**Figure 3A**). The lesion was then excised or contoured as planned (**Figures 3B**, **4A**) (6). Subsequently, either the PEEK implant or the FFF was placed and secured using procurved titanium plates (**Figures 3D**, **4C**). Concurrently, soft tissue defects were reconstructed using an appropriate flap (**Figures 3D**, **4C**). In the FFF group, the fibula was shaped intraoperatively using the osteotomy guide (**Figure 4B**). A drainage tube was placed before wound closure, and prophylactic antibiotics were administered during the perioperative period to prevent infection.

### 2.3 Statistical method

SPSS 21.0 was used for statistical analysis. The continuous variables were described by mean ± standard deviation after the normal distribution test and then compared for statistical differences between groups using an independent samples t-test. The categorical data were compared using Fisher’s exact test. A *p*-value of less than 0.05 was considered statistically significant.

## 3 Outcomes

### 3.1 Clinical features of patients

In this study, patients who underwent jaw reconstruction in the Department of Head and Neck Surgery of Zhejiang Provincial People’s Hospital between July 2019 and December 2023 were included. Among them, eight patients were treated with the FFF reconstruction, and eight received patient-specific PEEK implants. The FFF group consisted of six men and two women, with a mean age of 40.8 ± 21.2 years (range, 18–71 years). This group included two cases of maxillary defects and six cases of mandibular defects. Etiologies comprised four malignant and four benign lesions. All malignant cases underwent concurrent tumor resection, neck lymph node dissection, and FFF reconstruction. The PEEK group included five men and three women, with a mean age of 56.3 ± 12.6 years (range, 38–75 years). Two patients had maxillary defects, and six had mandibular defects. Five cases were malignant, and three were benign. Among these, four malignant and two benign cases underwent one-stage procedures involving lesion resection, placement of a PEEK implant, and—in malignant cases—concomitant lymph node dissection, along with soft tissue reconstruction using a flap. The remaining two cases received PEEK implant reconstruction in a second-stage operation.

A comparison of baseline characteristics between the two groups (**Table 2**) revealed no statistical differences in age, gender, tumor diameter, intraoperative blood loss, postoperative drainage volume, or reduction in BMI. However, the operative time was significantly shorter in the PEEK group compared to the FFF group. Whereas the FFF procedure required microvascular anastomosis, the PEEK approach also involved soft tissue reconstruction using either free or local flaps.

**Table 2:** Clinical and demographic profile of the FFF and 3D-printed PEEK cohorts.

Clinical parameters	FFF (n = 8)	PEEK (n = 8)	*P*
Age	40.8 ± 21.2	56.3 ± 12.6	0.1
Gender (male %)	75%	62.50%	0.5
Tumor diameter (mm)	35.4 ± 8.1	32.8 ± 11.3	0.6
Operation time (min)	545 ± 119.1	322.5 ± 158.6	0.007
Intraoperative bleeding (mL)	418.8 ± 385.4	212.5 ± 203.1	0.2
Postoperative drainage (mL)	625.0 ± 388.8	607.8 ± 480.7	0.9
ΔBMI (reduce)	1.48 ± 0.9	1.2 ± 1.3	0.6

FFF, free fibula flap; PEEK, polyether ether ketone.

### 3.2 Comparison of FFF and PEEK in benign or malignant tumors

The FFF group included four benign cases, while the PEEK group had three. A comparison between the two groups (**Table 3**) showed that patients in the PEEK group were significantly older than those in the FFF group, and the operative time was significantly shorter in the PEEK group. The mean values for intraoperative blood loss, postoperative drainage volume, and reduction in BMI were also lower in the PEEK group than in the FFF group. However, these differences did not reach statistical significance, likely due to the limited sample size. Among the malignant cases (**Table 4**), although the mean operative time remained shorter in the PEEK group compared to the FFF group, the difference was not statistically significant, which may also be attributed to the small number of patients. Furthermore, no significant differences were observed in gender, tumor diameter, intraoperative blood loss, postoperative drainage volume, or BMI reduction between the two groups in the malignant subgroup.

**Table 3:** Clinical characteristics of patients with benign jaw diseases reconstructed with FFF versus 3D-printed PEEK.

Clinical parameters	FFF (n = 4)	PEEK (n = 3)	*P*
Age	24 ± 4.2	50.7 ± 15.5	0.02
Gender (male %)	50%	33.30%	1
Tumor diameter (mm)	34.5 ± 6.7	23.0 ± 7.5	0.09
Operation time (min)	601.3 ± 25.0	231.7 ± 117.3	0.001
Intraoperative bleeding (mL)	612.5 ± 451.6	200 ± 200	0.21
Postoperative drainage (mL)	541.5 ± 384.0	191.7 ± 265.0	0.23
ΔBMI (reduce)	1.4 ± 0.7	0.9 ± 0.5	0.41

FFF, free fibula flap; PEEK, polyether ether ketone.

**Table 4:** Clinical characteristics of patients with malignant jaw diseases reconstructed with FFF versus 3D-printed PEEK.

Clinical parameters	FFF (n = 4)	PEEK (n = 5)	*P*
Age	57.5 ± 16.8	59.6 ± 11.0	0.8
Gender (male %)	100%	80.00%	1
Tumor diameter (mm)	36.3 ± 10.3	38.6 ± 9.0	0.7
Operation time (min)	488.8 ± 155.0	377.0 ± 165.0	0.3
Intraoperative bleeding (mL)	225.0 ± 206.2	220.0 ± 228.0	0.9
Postoperative drainage (mL)	708.5 ± 432.1	857.4 ± 402.0	0.6
ΔBMI (reduce)	1.6 ± 1.3	1.4 ± 1.7	0.8

FFF, free fibula flap; PEEK, polyether ether ketone.

### 3.3 Comparison of FFF and PEEK in mandibular reconstruction

Both the FFF and PEEK groups included six cases of mandibular reconstruction. A comparison of these cases (**Table 5**) revealed no significant differences in gender, tumor diameter, intraoperative blood loss, postoperative drainage volume, or reduction in BMI. However, patients in the PEEK group were significantly older than those in the FFF group, and the operative time was significantly shorter than that of the FFF group.

**Table 5:** Comparison of surgical outcomes in mandibular reconstruction with FFF versus 3D-printed PEEK.

Clinical parameters	FFF (n = 6)	PEEK (n = 6)	*P*
Age	37.8 ± 20.1	56.2 ± 9.3	0.07
Gender (male %)	83.3%	66.70%	1
Tumor diameter (mm)	36.3 ± 8.0	34.5 ± 12.7	0.7
Operation time (min)	559.2 ± 129.6	229.2 ± 202.6	0.007
Intraoperative bleeding (mL)	458.3 ± 443.2	183.3 ± 222.9	0.2
Postoperative drainage (mL)	627.8 ± 359.6	617.2 ± 437.1	1
ΔBMI (reduce)	1.4 ± 0.9	1.3 ± 1.5	0.9

FFF, free fibula flap; PEEK, polyether ether ketone.

### 3.4 Comparison of complications

All patients were successfully followed up (**Table 6**). In the FFF group, one case required the removal of the fixation titanium plate due to uncontrollable postoperative infection; however, the bone flap had already fused with the mandible, making further reconstruction unnecessary. Two patients experienced limited great toe movement, and one reported a persistent sensation of calf swelling. One case developed an occlusal disorder but maintained normal eating and speech function. Denture implantation was performed in two patients during the follow-up period. The remaining patients either used orthodontic braces or retained the tooth defects due to factors such as bone resorption, leading to insufficient bone thickness or unsatisfactory healing, resulting in poor stability.

**Table 6:** Comparison of postoperative complications between the FFF and PEEK groups.

Groups	Postoperative bleeding		Uncontrollable infection		Alternative operation		Occlusal		Dysphagia		Linguistic function		Calf stability		Dental implant	
	Y	N	Y	N	Y	N	Y	N	Y	N	Y	N	Y	N	Y	N
FFF	0	8	1	7	0	8	7	1	0	8	8	0	5	3	2	6
PEEK	1	7	2	6	2	6	6	2	1	7	8	0	7	1	0	8

Y, yes; N, no; FFF, free fibula flap; PEEK, polyether ether ketone.

In the PEEK group, one patient required debridement due to postoperative bleeding. Two patients developed uncontrolled infections, which necessitated the removal of both the PEEK implant and the fixation titanium plate. After the infection was controlled, both cases underwent secondary reconstruction using the FFF approach. One of these patients subsequently experienced poor calf stability. Another case was diagnosed with dysphagia, which was attributed to radiotherapy rather than the PEEK implantation. None of the patients in this group received dental implants.

## 4 Discussion

### 4.1 Limitations of free fibula flap reconstruction

The FFF represents a well-established approach for jaw reconstruction, offering the distinct advantage of simultaneous bone and soft tissue restoration (7). However, this technique is associated with donor-site morbidity, including complications such as chronic pain, infection, sensory deficits, and impaired lower limb function. Additionally, the recipient site may also experience adverse events, including infection, titanium plate exposure, bone absorption, and impaired bone healing (8). Furthermore, the inherent contour limitations of the fibula often result in a less anatomical reconstruction of the jaw, typically necessitating complex shaping and prolonged operative time (9, 10). The extended duration of surgery concomitantly increases anesthesia exposure. The frequent use of vascular staplers during the procedure may further elevate overall treatment costs. Consequently, there has been growing interest in developing alternative reconstructive strategies to overcome these limitations of the FFF technique (11, 12).

### 4.2 PEEK represents a clinically relevant alternative to the free fibula flap

PEEK has been widely adopted in orthopedic bone repair due to its favorable properties, including an elastic modulus similar to human bone, high mechanical strength, biocompatibility, radiolucency, and excellent plasticity (13). Building on these advantages, this study utilized 3D printing technology to fabricate patient-specific PEEK implants that precisely fit the jaw defect, thereby eliminating the need for fibula harvest and associated donor-site morbidity. The use of PEEK implants also reduced operative time and consequently decreased anesthesia-related risks. However, the PEEK implants used in this study were not designed to support dental implantation, making FFF a more suitable option for younger people who tend to seek functional dental rehabilitation. Among the eight cases in the PEEK group, two required explantation due to uncontrolled infection, with successful salvage reconstruction subsequently achieved using FFF after infection control. These outcomes underscore infection as a primary cause of PEEK reconstruction failure, highlighting the need to improve the anti-infection properties of PEEK-based materials to increase clinical success (14). Several other limitations must also be considered. The high material cost, inability to support dental implantation in the reconstructed segment (compromising long-term masticatory function), and the inherent risk of infection that may necessitate implant removal all restrict its broader application.

Additionally, the intraoperative adaptability of preformed PEEK implants is limited in case of unexpected surgical findings, frequently necessitating complementary soft tissue reconstruction with flaps. Finally, the successful implementation of 3D-printed PEEK jaw reconstruction demands a high level of technical expertise and a coordinated multidisciplinary team, further constraining its use in general practice (15).

### 4.3 Limitations of this study

A key limitation of this study is the small sample size, which may limit the generalizability of the findings regarding the application of PEEK in jaw reconstruction. Nevertheless, successful long-term reconstruction was achieved in six out of the eight patients in the PEEK group, allowing these individuals to avoid donor-site morbidity in the lower limb, reduce operative time, and decrease anesthetic exposure. In two cases within the PEEK group, reconstruction was converted to FFF following uncontrolled infection, suggesting that FFF remains a viable salvage option when PEEK-based reconstruction fails. Another constraint lies in the inherent limitations of PEEK material, including its bio-inertness, which may hinder osseointegration and bone remodeling, as well as its limited antibacterial properties, often necessitating implant removal in cases of infection. On a positive note, increasing studies have shown that the high plasticity of PEEK enables various structural and compositional modifications, offering promising avenues to overcome these limitations in the future (14). Furthermore, it should be noted that heterogeneity in pathology and its associated treatments, such as radiotherapy, may have acted as confounding factors. Additional limitations include the retrospective, single-center design and the relatively short follow-up period, which restrict the analysis primarily to short-term outcomes and comparisons between two groups. The rarity of these patients also precludes sufficiently powerful subgroup analyses. Therefore, this study serves to provide preliminary data supporting future refinements of PEEK as a potential alternative to FFF, rather than establishing a definitive treatment protocol.

### 4.4 The critical role of complication management

Complications play a critical role in determining the long-term success of jaw reconstruction. Autogenous bone grafts, while commonly used, are associated with issues such as bone resorption that may compromise repair quality, in addition to the unavoidable donor-site morbidity (16). Allogeneic bone offers another alternative for jaw reconstruction, yet its application is constrained by immunological rejection and limited donor availability (17, 18). Titanium alloy, particularly with the advent of 3D printing technology, has enabled the precise reconstruction of mandibular defects. However, complications such as implant fracture, corrosion, screw loosening, plate exposure, and peri-implant bone absorption can lead to reconstruction failure over the long term (19). An ideal jaw reconstruction material should possess some properties such as high biocompatibility, controllable degradation, and excellent manufacturability. Emerging biomaterials are being designed with specialized microstructures that enhance osteogenesis and angiogenesis, thereby reducing the risk of non-union. Furthermore, the incorporation of antibacterial agents into these materials shows promise in mitigating the incidence of refractory infections (20).

### 4.5 Future perspectives and research horizons of jaw reconstruction

Utilizing 3D printing technology, PEEK enables the precise reconstruction of jaw defects, offering accurate anatomical restoration (21). However, its broader application remains constrained by certain complications. Capitalizing on the high plasticity of PEEK, it is possible to enhance its bioactivity—without compromising its physical properties—through structural modifications and the incorporation of bioactive molecules. These strategies can improve the adhesion and proliferation of jaw-derived mesenchymal stem cells, thereby facilitating osteogenic integration after reconstruction (22–24). Infection is a major determinant of the success of PEEK implantation. Pretreatment approaches, such as coating the implant surface with silver nanoparticles (AgNPs), can significantly enhance antibacterial properties, inhibit microbial colonization, and reduce implant failure due to infection (25). Beyond anatomical reconstruction, future efforts should also address the restoration of neurological continuity, sensory recovery, and functional rehabilitation of facial expression (26, 27). In conclusion, the findings of this study indicate that 3D-printed PEEK implants hold considerable promise for jaw reconstruction, provided that limitations related to bioactivity and infection are systematically addressed.

## Data Availability

Publicly available datasets were analyzed in this study. This data can be found here: https://pan.baidu.com/s/1wMVtRflUqu2E9MRc8m7pgQ?pwd=9ag4 Extraction code: 9ag4.

## References

[1] Järvinen S Suojanen J Kormi E Wilkman T Kiukkonen A Leikola J. The use of patient specific polyetheretherketone implants for reconstruction of maxillofacial deformities. J Craniomaxillofac Surg. (2019) 47:1072–6. doi: 10.1016/j.jcms.2019.03.018, PMID: 31103433

[2] Aftabi H Zaraska K Eghbal A McGregor S Prisman E Hodgson A. Computational models and their applications in biomechanical analysis of mandibular reconstruction surgery. Comput Biol Med. (2024) 169:107887. doi: 10.1016/j.compbiomed.2023.107887, PMID: 38160502

[3] Yu D Lei X Zhu H. Modification of polyetheretherketone (PEEK) physical features to improve osteointegration. J Zhejiang Univ Sci B. (2022) 23:189–203. doi: 10.1631/jzus.B2100622, PMID: 35261215 PMC8913926

[4] Oladapo BI Zahedi SA Ismail SO Omigbodun FT. 3D printing of PEEK and its composite to increase biointerfaces as a biomedical material- A review. Colloids Surf B Biointerfaces. (2021) 203:111726. doi: 10.1016/j.colsurfb.2021.111726, PMID: 33865088

[5] Cho KH Papay FA Yanof J West K Bassiri Gharb B Rampazzo A. Mixed reality and 3D printed models for planning and execution of face transplantation. Ann Surg. (2021) 274:e1238–e46. doi: 10.1097/SLA.0000000000003794, PMID: 32224738

[6] Xu J Lai F Liu Y Tan Z Zheng C Wang J. Novel computer-aided reconstruction of soft tissue defects following resection of oral and oropharyngeal squamous cell carcinoma. World J Surg Oncol. (2022) 20:196. doi: 10.1186/s12957-022-02654-7, PMID: 35698194 PMC9195432

[7] Gao N Fu K Cai J Chen H He W. The role of folded fibular flap in patients’ reconstruction of mandibular defects: a retrospective clinical study. Sci Rep. (2021) 11:23853. doi: 10.1038/s41598-021-03331-7, PMID: 34903811 PMC8668899

[8] Liu AQ Deane EC Heffernan A Ji Y Durham JS Prisman E. Patient-reported outcomes and morbidity after head and neck reconstructions: An evaluation of fibular and scapular free flaps. Oral Oncol. (2022) 132:106019. doi: 10.1016/j.oraloncology.2022.106019, PMID: 35841704

[9] Ni Y Zhang X Meng Z Li Z Li S Xu ZF. Digital navigation and 3D model technology in mandibular reconstruction with fibular free flap: A comparative study. J Stomatol Oral Maxillofac Surg. (2021) 122:e59–64. doi: 10.1016/j.jormas.2020.11.002, PMID: 33242657

[10] Liu YB Wu D Wang JY Lun XH Dai W. Meta-analysis of the survival rate and postoperative infection rate of primary and secondary implants after vascularized fibula transplantation for reconstruction of jaw defects. Int J Implant Dent. (2023) 9:51. doi: 10.1186/s40729-023-00514-x, PMID: 38108942 PMC10728391

[11] Zhao J Zhou YH Zhao YQ Gao ZR Ouyang ZY Ye Q. Oral cavity-derived stem cells and preclinical models of jaw-bone defects for bone tissue engineering. Stem Cell Res Ther. (2023) 14:39. doi: 10.1186/s13287-023-03265-z, PMID: 36927449 PMC10022059

[12] Hurley CM McConn Walsh R Shine NP O’Neill JP Martin F O’Sullivan JB. Current trends in craniofacial reconstruction. Surgeon. (2023) 21:e118–e25. doi: 10.1016/j.surge.2022.04.004, PMID: 35525818

[13] Dondani JR Iyer J Tran SD. Surface treatments of PEEK for osseointegration to bone. Biomolecules. (2023) 13. doi: 10.3390/biom13030464, PMID: 36979399 PMC10046336

[14] Zheng Z Liu P Zhang X Jingguo X Yongjie W Zou X. Strategies to improve bioactive and antibacterial properties of polyetheretherketone (PEEK) for use as orthopedic implants. Mater Today Bio. (2022) 16:100402. doi: 10.1016/j.mtbio.2022.100402, PMID: 36105676 PMC9466655

[15] Le Donne M Jouan R Bourlet J Louvrier A Ducret M Sigaux N. Inferior alveolar nerve allogenic repair following mandibulectomy: A systematic review. J Stomatol Oral Maxillofac Surg. (2022) 123:233–8. doi: 10.1016/j.jormas.2021.04.007, PMID: 33933668

[16] Zhang Q Wu W Qian C Xiao W Zhu H Guo J. Advanced biomaterials for repairing and reconstruction of mandibular defects. Mater Sci Eng C Mater Biol Appl. (2019) 103:109858. doi: 10.1016/j.msec.2019.109858, PMID: 31349473

[17] Özkan Ö Özkan Ö Doğan U Yılmaz VT Uysal H Ündar L. Consideration of difficulties and exit strategies in a case of face allotransplantation resulting in failure. Microsurgery. (2017) 37:661–8. doi: 10.1002/micr.30137, PMID: 28493355

[18] Stopa Z Siewert-Gutowska M Abed K Szubińska-Lelonkiewicz D Kamiński A Fiedor P. Evaluation of the safety and clinical efficacy of allogeneic bone grafts in the reconstruction of the maxilla and mandible. Transplant Proc. (2018) 50:2199–201. doi: 10.1016/j.transproceed.2018.02.122, PMID: 30177136

[19] Xue R Lai Q Xing H Zhong C Zhao Y Zhu K. Finite element analysis and clinical application of 3D-printed Ti alloy implant for the reconstruction of mandibular defects. BMC Oral Health. (2024) 24:95. doi: 10.1186/s12903-024-03857-y, PMID: 38233785 PMC10792868

[20] Guo J Yao H Li X Chang L Wang Z Zhu W. Advanced Hydrogel systems for mandibular reconstruction. Bioact Mater. (2023) 21:175–93. doi: 10.1016/j.bioactmat.2022.08.001, PMID: 36093328 PMC9413641

[21] Truscott A Zamani R Akrami M. Comparing the use of conventional and three-dimensional printing (3DP) in mandibular reconstruction. BioMed Eng Online. (2022) 21:18. doi: 10.1186/s12938-022-00989-6, PMID: 35305669 PMC8934485

[22] Ferroni L D’Amora U Leo S Tremoli E Raucci MG Ronca A. PEEK and hyaluronan-based 3D printed structures: promising combination to improve bone regeneration. Molecules. (2022) 27. doi: 10.3390/molecules27248749, PMID: 36557882 PMC9787780

[23] Cao SS Li SY Geng YM Kapat K Liu SB Perera FH. Prefabricated 3D-printed tissue-engineered bone for mandibular reconstruction: A preclinical translational study in primate. ACS Biomater Sci Eng. (2021) 7:5727–38. doi: 10.1021/acsbiomaterials.1c00509, PMID: 34808042 PMC8672350

[24] Emam H Leach D Sun Z Tee BC Karatas B Kim DG. The effect of parathyroid hormone analogues when added to mineralized bone xenografts. J Oral Implantol. (2020) 46:372–9. doi: 10.1563/aaid-joi-D-19-00016, PMID: 32299092

[25] Deng L Deng Y Xie K. AgNPs-decorated 3D printed PEEK implant for infection control and bone repair. Colloids Surf B Biointerfaces. (2017) 160:483–92. doi: 10.1016/j.colsurfb.2017.09.061, PMID: 28992487

[26] Kaplan J Lee ZH Grome L Yao C Mericli AF Roubaud MS. Sensory outcomes for inferior alveolar nerve reconstruction with allograft following free fibula mandible reconstruction. Plast Reconstr Surg. (2023) 152:499e–506e. doi: 10.1097/PRS.0000000000010286, PMID: 36780351

[27] Chen LW Lakhiani C Huang JJ Abdelrahman M Huang SF Chang TN. Holistic reconstruction of mandible defect, lower lip and chin sensation, and smile reanimation in an advanced gum cancer patient: A case report. Microsurgery. (2021) 41:361–5. doi: 10.1002/micr.30684, PMID: 33185301

